# {1,1′-[2,2-Dimethyl­propane-1,3-diylbis(nitrilo­methyl­idyne)]di-2-naphtholato}nickel(II)

**DOI:** 10.1107/S1600536810007373

**Published:** 2010-03-03

**Authors:** Hadi Kargar, Reza Kia, Islam Ullah Khan, Karim Zare

**Affiliations:** a Department of Chemistry, School of Science, Payame Noor University (PNU), Ardakan, Yazd, Iran; b Department of Chemistry, Science and Research Branch, Islamic Azad University, Tehran, Iran; c Materials Chemistry Laboratory, Department of Chemistry, GC University, Lahore 54000, Pakistan

## Abstract

In the title Schiff base complex, [Ni(C_27_H_24_N_2_O_2_)], the Ni^II^ atom shows a slightly distorted square-planar geometry. The dihedral angle between the mean planes of the two aromatic rings is 6.16 (6)°. In the crystal, pairs of inter­molecular weak C—H⋯O hydrogen bonds link neighboring mol­ecules into a chain along the *a* axis. The crystal structure is further stabilized by two inter­molecular π–π inter­actions with centroid–centroid distances of 3.7252 (13) and 3.8323 (13) Å.

## Related literature

For bond-length data, see: Allen *et al.* (1987 ▶). For background to Schiff base–metal complexes, see: Granovski *et al.* (1993 ▶); Blower *et al.* (1998 ▶); Elmali *et al.* (2000 ▶).
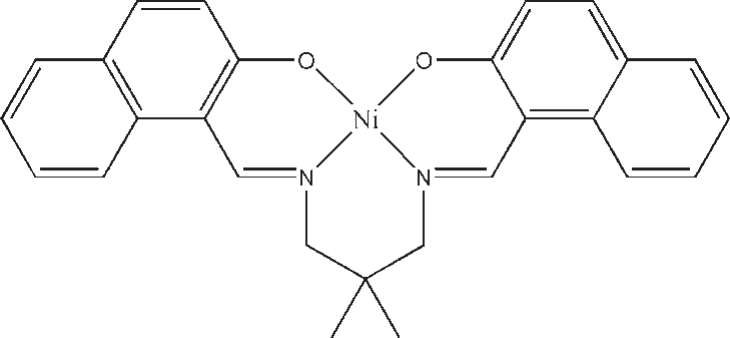


## Experimental

### 

#### Crystal data

[Ni(C_27_H_24_N_2_O_2_)]
*M* *_r_* = 467.19
Triclinic, 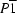

*a* = 7.5016 (2) Å
*b* = 11.3583 (3) Å
*c* = 13.0173 (3) Å
α = 87.846 (2)°
β = 89.496 (1)°
γ = 76.196 (1)°
*V* = 1076.35 (5) Å^3^
*Z* = 2
Mo *K*α radiation
μ = 0.93 mm^−1^
*T* = 296 K
0.45 × 0.23 × 0.12 mm

#### Data collection

Bruker SMART APEXII CCD area-detector diffractometer
Absorption correction: multi-scan (*SADABS*; Bruker, 2005 ▶) *T*_min_ = 0.680, *T*_max_ = 0.897
23454 measured reflections
5293 independent reflections
3802 reflections with *I* > 2σ(*I*)
*R*_int_ = 0.034

#### Refinement

*R*[*F* ^2^ > 2σ(*F* ^2^)] = 0.033
*wR*(*F* ^2^) = 0.085
*S* = 1.02
5293 reflections
289 parameters
H-atom parameters constrained
Δρ_max_ = 0.23 e Å^−3^
Δρ_min_ = −0.28 e Å^−3^

### 

Data collection: *APEX2* (Bruker, 2005 ▶); cell refinement: *SAINT* (Bruker, 2005 ▶); data reduction: *SAINT*; program(s) used to solve structure: *SHELXTL* (Sheldrick, 2008 ▶); program(s) used to refine structure: *SHELXTL*; molecular graphics: *SHELXTL*; software used to prepare material for publication: *SHELXTL* and *PLATON* (Spek, 2009 ▶).

## Supplementary Material

Crystal structure: contains datablocks global, I. DOI: 10.1107/S1600536810007373/bq2198sup1.cif

Structure factors: contains datablocks I. DOI: 10.1107/S1600536810007373/bq2198Isup2.hkl

Additional supplementary materials:  crystallographic information; 3D view; checkCIF report

## Figures and Tables

**Table:** 

Ni1—O2	1.8453 (12)
Ni1—O1	1.8501 (12)
Ni1—N1	1.8617 (14)
Ni1—N2	1.8632 (14)

**Table:** 

O2—Ni1—O1	84.48 (5)
O1—Ni1—N1	92.08 (6)
O2—Ni1—N2	92.02 (6)
N1—Ni1—N2	91.51 (6)

**Table 2:** Hydrogen-bond geometry (Å, °)

*D*—H⋯*A*	*D*—H	H⋯*A*	*D*⋯*A*	*D*—H⋯*A*
C12—H12*B*⋯O1^i^	0.97	2.46	3.422 (2)	171
C14—H14*B*⋯O2^ii^	0.97	2.54	3.502 (2)	170

Symmetry codes: (i) 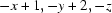; (ii) 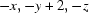.

**Fig. 1.**
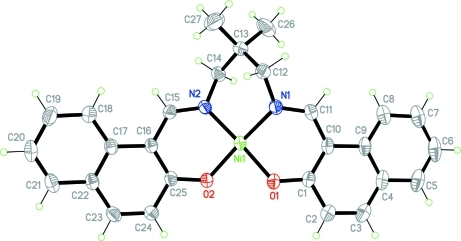
The asymmetric unit of the title compound, showing 50% probability displacement ellipsoids and the atomic numbering.

**Fig. 2.**
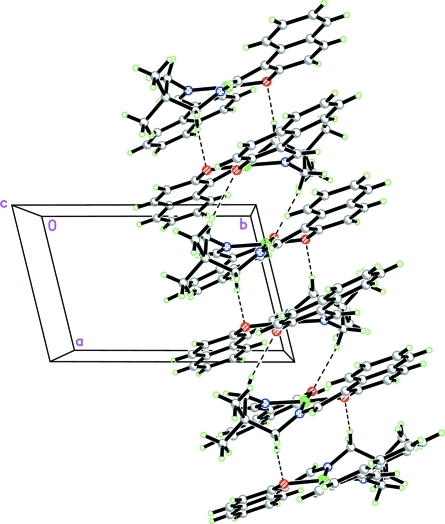
The packing diagram of the title compound, showing a 1-D infinite chain along the a-axis by the intermolecular C—H···O interactions. The intermolecular h-bonds are shown as dashed lines.

## supplementary crystallographic information

### Comment

Schiff base complexes are one of the most important stereochemical models in transition metal coordination chemistry, with the ease of preparation and structural variations (Granovski et al., 1993). Metal derivatives of the Schiff bases have been studied extensively, and Ni(II) and Cu(II) complexes play a major role in both synthetic and structural research (Elmali et al., 2000; Blower et al., 1998). The asymmetric unit of the title compound, Fig. 1, comprises one unit of the Schiff base complex. The bond lengths (Allen et al., 1987) and angles are within the normal ranges. The geometry around the Ni(II) atom is a slightly distorted square-planar which is coordinated by the N_2_O_2_ donor atoms of the desired potentially tetradenate Schiff base ligand (Table 1). Pairs of weak intermolecular C—H···O hydrogen bonds (Table 2) link neighboring molecules into a chain along the a-axis. The dihedral angle between the mean planes of the two aromatic rings is 6.16 (6)°. The crystal structure is further stabilized by the intermolecular π–π interactions [Cg1···Cg2^i^ = 3.7252 (13) and Cg1···Cg2^ii^ = 3.8323 (13) Å; Cg1 and Cg2 are the centroids of the C4/C5/C6/C7/C8/C9 and C17/C18/C19/C20/C21/C22 benzene rings].

### Experimental

The title compound was synthesized by adding bis(naphthaldiminato)-2,3- propanediamine (2 mmol) to a solution of NiCl_2_. 6 H_2_O (2 mmol) in ethanol (30 ml). The mixture was refluxed with stirring for half an hour. The resultant red solution was filtered. Red single crystals of the title compound suitable for *X*-ray structure determination were recrystallized from ethanol by slow evaporation of the solvents at room temperature over several days.

### Refinement

All hydrogen atoms were positioned geometrically with C-H = 0.93-0.97 Å and included in a riding model approximation with U_iso_ (H) = 1.2 U_eq_ (C).

### Crystal data

**Table:**

[Ni(C_27_H_24_N_2_O_2_)]	*Z* = 2
*M**_r_* = 467.19	*F*(000) = 488
Triclinic, *P*1	*D*_x_ = 1.442 Mg m^−^^3^
Hall symbol: -P 1	Mo *K*α radiation, λ = 0.71073 Å
*a* = 7.5016 (2) Å	Cell parameters from 7107 reflections
*b* = 11.3583 (3) Å	θ = 2.4–28.2°
*c* = 13.0173 (3) Å	µ = 0.93 mm^−^^1^
α = 87.846 (2)°	*T* = 296 K
β = 89.496 (1)°	Block, red
γ = 76.196 (1)°	0.45 × 0.23 × 0.12 mm
*V* = 1076.35 (5) Å^3^	

### Data collection

**Table:**

Bruker SMART APEXII CCD area-detector diffractometer	5293 independent reflections
Radiation source: fine-focus sealed tube	3802 reflections with *I* > 2σ(*I*)
graphite	*R*_int_ = 0.034
φ and ω scans	θ_max_ = 28.3°, θ_min_ = 2.5°
Absorption correction: multi-scan (*SADABS*; Bruker, 2005)	*h* = −9→10
*T*_min_ = 0.680, *T*_max_ = 0.897	*k* = −15→15
23454 measured reflections	*l* = −17→17

### Refinement

**Table:**

Refinement on *F*^2^	Primary atom site location: structure-invariant direct methods
Least-squares matrix: full	Secondary atom site location: difference Fourier map
*R*[*F*^2^ > 2σ(*F*^2^)] = 0.033	Hydrogen site location: inferred from neighbouring sites
*wR*(*F*^2^) = 0.085	H-atom parameters constrained
*S* = 1.02	*w* = 1/[σ^2^(*F*_o_^2^) + (0.0358*P*)^2^ + 0.2515*P*] where *P* = (*F*_o_^2^ + 2*F*_c_^2^)/3
5293 reflections	(Δ/σ)_max_ = 0.002
289 parameters	Δρ_max_ = 0.23 e Å^−^^3^
0 restraints	Δρ_min_ = −0.28 e Å^−^^3^

### Special details

**Table:**

Geometry. All esds (except the esd in the dihedral angle between two l.s. planes) are estimated using the full covariance matrix. The cell esds are taken into account individually in the estimation of esds in distances, angles and torsion angles; correlations between esds in cell parameters are only used when they are defined by crystal symmetry. An approximate (isotropic) treatment of cell esds is used for estimating esds involving l.s. planes.
Refinement. Refinement of F^2^ against ALL reflections. The weighted R-factor wR and goodness of fit S are based on F^2^, conventional R-factors R are based on F, with F set to zero for negative F^2^. The threshold expression of F^2^ > 2sigma(F^2^) is used only for calculating R-factors(gt) etc. and is not relevant to the choice of reflections for refinement. R-factors based on F^2^ are statistically about twice as large as those based on F, and R- factors based on ALL data will be even larger.

### Fractional atomic coordinates and isotropic or equivalent isotropic displacement parameters (Å^2^)

**Table:**

	*x*	*y*	*z*	*U*_iso_*/*U*_eq_	
Ni1	0.25048 (3)	1.00150 (2)	−0.002820 (16)	0.03427 (8)	
O1	0.22183 (18)	1.16579 (11)	0.01506 (9)	0.0429 (3)	
O2	0.20302 (18)	1.04717 (11)	−0.13928 (9)	0.0417 (3)	
N1	0.30698 (19)	0.96456 (13)	0.13543 (11)	0.0351 (3)	
N2	0.26661 (19)	0.83899 (13)	−0.02841 (10)	0.0337 (3)	
C1	0.1646 (2)	1.22188 (16)	0.09929 (14)	0.0376 (4)	
C2	0.1041 (3)	1.35083 (18)	0.09098 (15)	0.0473 (5)	
H2A	0.1129	1.3909	0.0282	0.057*	
C3	0.0340 (3)	1.41573 (19)	0.17329 (17)	0.0528 (5)	
H3A	−0.0049	1.4997	0.1655	0.063*	
C4	0.0180 (3)	1.35973 (19)	0.27082 (15)	0.0468 (5)	
C5	−0.0599 (3)	1.4277 (2)	0.35586 (19)	0.0611 (6)	
H5A	−0.1016	1.5114	0.3481	0.073*	
C6	−0.0747 (3)	1.3721 (3)	0.44875 (19)	0.0672 (7)	
H6A	−0.1248	1.4179	0.5043	0.081*	
C7	−0.0150 (3)	1.2469 (3)	0.46062 (16)	0.0607 (6)	
H7A	−0.0265	1.2091	0.5241	0.073*	
C8	0.0610 (3)	1.1785 (2)	0.37951 (15)	0.0503 (5)	
H8A	0.0988	1.0947	0.3887	0.060*	
C9	0.0826 (2)	1.23247 (18)	0.28295 (14)	0.0410 (4)	
C10	0.1613 (2)	1.16358 (17)	0.19568 (13)	0.0365 (4)	
C11	0.2568 (2)	1.03958 (17)	0.20838 (13)	0.0373 (4)	
H11A	0.2857	1.0099	0.2752	0.045*	
C12	0.4315 (2)	0.84660 (16)	0.15985 (14)	0.0395 (4)	
H12A	0.4724	0.8452	0.2305	0.047*	
H12B	0.5387	0.8369	0.1159	0.047*	
C13	0.3433 (3)	0.73967 (16)	0.14614 (14)	0.0402 (4)	
C14	0.2095 (3)	0.76569 (17)	0.05547 (13)	0.0405 (4)	
H14A	0.1980	0.6893	0.0288	0.049*	
H14B	0.0896	0.8079	0.0798	0.049*	
C15	0.3060 (2)	0.79094 (16)	−0.11691 (13)	0.0357 (4)	
H15A	0.3179	0.7077	−0.1198	0.043*	
C16	0.3326 (2)	0.85414 (16)	−0.21032 (13)	0.0334 (4)	
C17	0.4069 (2)	0.78886 (16)	−0.30034 (13)	0.0349 (4)	
C18	0.4910 (3)	0.66487 (18)	−0.29783 (15)	0.0467 (5)	
H18A	0.5003	0.6210	−0.2355	0.056*	
C19	0.5602 (3)	0.6063 (2)	−0.38503 (17)	0.0543 (5)	
H19A	0.6147	0.5236	−0.3810	0.065*	
C20	0.5499 (3)	0.6689 (2)	−0.47890 (16)	0.0553 (6)	
H20A	0.5966	0.6284	−0.5377	0.066*	
C21	0.4713 (3)	0.7895 (2)	−0.48451 (15)	0.0490 (5)	
H21A	0.4645	0.8314	−0.5477	0.059*	
C22	0.3996 (2)	0.85265 (17)	−0.39621 (13)	0.0385 (4)	
C23	0.3185 (3)	0.97905 (18)	−0.40085 (14)	0.0437 (4)	
H23A	0.3093	1.0209	−0.4641	0.052*	
C24	0.2541 (3)	1.04042 (17)	−0.31591 (13)	0.0418 (4)	
H24A	0.2027	1.1234	−0.3219	0.050*	
C25	0.2638 (2)	0.98001 (16)	−0.21745 (13)	0.0345 (4)	
C26	0.2337 (3)	0.7194 (2)	0.24235 (16)	0.0643 (6)	
H26A	0.1385	0.7910	0.2535	0.096*	
H26B	0.3141	0.7027	0.3008	0.096*	
H26C	0.1798	0.6519	0.2330	0.096*	
C27	0.4978 (3)	0.6277 (2)	0.1266 (2)	0.0680 (7)	
H27A	0.5647	0.6425	0.0662	0.102*	
H27B	0.4465	0.5592	0.1168	0.102*	
H27C	0.5793	0.6113	0.1846	0.102*	

### Atomic displacement parameters (Å^2^)

**Table:**

	*U*^11^	*U*^22^	*U*^33^	*U*^12^	*U*^13^	*U*^23^
Ni1	0.04204 (14)	0.03339 (13)	0.02735 (13)	−0.00840 (9)	0.00091 (9)	−0.00494 (9)
O1	0.0599 (8)	0.0363 (7)	0.0329 (7)	−0.0114 (6)	0.0073 (6)	−0.0074 (5)
O2	0.0568 (8)	0.0342 (7)	0.0299 (6)	−0.0021 (6)	0.0002 (6)	−0.0039 (5)
N1	0.0380 (8)	0.0368 (8)	0.0313 (8)	−0.0099 (7)	0.0004 (6)	−0.0037 (6)
N2	0.0391 (8)	0.0354 (8)	0.0276 (7)	−0.0105 (6)	−0.0005 (6)	−0.0014 (6)
C1	0.0387 (9)	0.0386 (10)	0.0374 (10)	−0.0116 (8)	0.0002 (7)	−0.0094 (8)
C2	0.0562 (12)	0.0402 (11)	0.0469 (11)	−0.0134 (9)	0.0001 (9)	−0.0071 (9)
C3	0.0546 (12)	0.0410 (11)	0.0629 (14)	−0.0090 (10)	−0.0027 (10)	−0.0165 (10)
C4	0.0404 (10)	0.0527 (12)	0.0483 (12)	−0.0096 (9)	0.0003 (8)	−0.0236 (10)
C5	0.0497 (12)	0.0629 (15)	0.0685 (16)	−0.0042 (11)	0.0026 (11)	−0.0362 (13)
C6	0.0504 (13)	0.096 (2)	0.0538 (15)	−0.0083 (13)	0.0074 (10)	−0.0419 (14)
C7	0.0506 (12)	0.091 (2)	0.0405 (12)	−0.0154 (13)	0.0052 (9)	−0.0209 (12)
C8	0.0456 (11)	0.0685 (14)	0.0376 (11)	−0.0138 (10)	0.0017 (8)	−0.0140 (10)
C9	0.0319 (9)	0.0539 (12)	0.0391 (10)	−0.0115 (8)	−0.0010 (7)	−0.0177 (9)
C10	0.0355 (9)	0.0435 (10)	0.0329 (9)	−0.0125 (8)	0.0004 (7)	−0.0113 (8)
C11	0.0387 (9)	0.0454 (11)	0.0294 (9)	−0.0125 (8)	−0.0002 (7)	−0.0048 (8)
C12	0.0404 (10)	0.0402 (10)	0.0363 (10)	−0.0063 (8)	−0.0045 (8)	−0.0014 (8)
C13	0.0485 (11)	0.0368 (10)	0.0352 (10)	−0.0102 (9)	−0.0012 (8)	0.0023 (8)
C14	0.0496 (11)	0.0424 (10)	0.0336 (10)	−0.0193 (9)	0.0020 (8)	−0.0008 (8)
C15	0.0395 (9)	0.0326 (9)	0.0360 (10)	−0.0098 (8)	−0.0021 (7)	−0.0038 (8)
C16	0.0348 (9)	0.0371 (10)	0.0294 (9)	−0.0100 (8)	−0.0010 (7)	−0.0043 (7)
C17	0.0334 (9)	0.0414 (10)	0.0317 (9)	−0.0118 (8)	−0.0014 (7)	−0.0060 (8)
C18	0.0531 (12)	0.0439 (11)	0.0412 (11)	−0.0070 (9)	0.0023 (9)	−0.0063 (9)
C19	0.0575 (13)	0.0458 (12)	0.0558 (14)	−0.0030 (10)	0.0071 (10)	−0.0158 (10)
C20	0.0583 (13)	0.0654 (15)	0.0435 (12)	−0.0148 (11)	0.0142 (10)	−0.0235 (11)
C21	0.0558 (12)	0.0631 (14)	0.0321 (10)	−0.0212 (11)	0.0073 (9)	−0.0093 (9)
C22	0.0397 (10)	0.0471 (11)	0.0313 (9)	−0.0148 (8)	0.0004 (7)	−0.0075 (8)
C23	0.0534 (11)	0.0492 (12)	0.0297 (9)	−0.0150 (9)	−0.0035 (8)	0.0023 (8)
C24	0.0522 (11)	0.0379 (10)	0.0343 (10)	−0.0087 (9)	−0.0045 (8)	−0.0003 (8)
C25	0.0372 (9)	0.0373 (10)	0.0297 (9)	−0.0093 (8)	−0.0022 (7)	−0.0054 (7)
C26	0.0814 (16)	0.0800 (17)	0.0403 (12)	−0.0387 (14)	0.0001 (11)	0.0106 (11)
C27	0.0766 (16)	0.0431 (13)	0.0787 (17)	−0.0029 (12)	−0.0123 (13)	−0.0017 (11)

### Geometric parameters (Å, °)

**Table:**

Ni1—O2	1.8453 (12)	C12—H12B	0.9700
Ni1—O1	1.8501 (12)	C13—C27	1.530 (3)
Ni1—N1	1.8617 (14)	C13—C14	1.530 (2)
Ni1—N2	1.8632 (14)	C13—C26	1.532 (3)
O1—C1	1.306 (2)	C14—H14A	0.9700
O2—C25	1.308 (2)	C14—H14B	0.9700
N1—C11	1.293 (2)	C15—C16	1.426 (2)
N1—C12	1.464 (2)	C15—H15A	0.9300
N2—C15	1.295 (2)	C16—C25	1.399 (2)
N2—C14	1.470 (2)	C16—C17	1.446 (2)
C1—C10	1.399 (3)	C17—C18	1.398 (3)
C1—C2	1.426 (3)	C17—C22	1.414 (2)
C2—C3	1.354 (3)	C18—C19	1.372 (3)
C2—H2A	0.9300	C18—H18A	0.9300
C3—C4	1.416 (3)	C19—C20	1.384 (3)
C3—H3A	0.9300	C19—H19A	0.9300
C4—C9	1.414 (3)	C20—C21	1.356 (3)
C4—C5	1.414 (3)	C20—H20A	0.9300
C5—C6	1.359 (3)	C21—C22	1.411 (2)
C5—H5A	0.9300	C21—H21A	0.9300
C6—C7	1.388 (3)	C22—C23	1.419 (3)
C6—H6A	0.9300	C23—C24	1.353 (2)
C7—C8	1.372 (3)	C23—H23A	0.9300
C7—H7A	0.9300	C24—C25	1.425 (2)
C8—C9	1.403 (3)	C24—H24A	0.9300
C8—H8A	0.9300	C26—H26A	0.9600
C9—C10	1.444 (2)	C26—H26B	0.9600
C10—C11	1.424 (3)	C26—H26C	0.9600
C11—H11A	0.9300	C27—H27A	0.9600
C12—C13	1.532 (2)	C27—H27B	0.9600
C12—H12A	0.9700	C27—H27C	0.9600
			
O2—Ni1—O1	84.48 (5)	C14—C13—C12	110.48 (14)
O2—Ni1—N1	175.84 (6)	C27—C13—C26	110.90 (18)
O1—Ni1—N1	92.08 (6)	C14—C13—C26	107.42 (16)
O2—Ni1—N2	92.02 (6)	C12—C13—C26	110.25 (16)
O1—Ni1—N2	175.83 (6)	N2—C14—C13	113.35 (14)
N1—Ni1—N2	91.51 (6)	N2—C14—H14A	108.9
C1—O1—Ni1	125.17 (12)	C13—C14—H14A	108.9
C25—O2—Ni1	125.40 (11)	N2—C14—H14B	108.9
C11—N1—C12	118.80 (15)	C13—C14—H14B	108.9
C11—N1—Ni1	124.30 (13)	H14A—C14—H14B	107.7
C12—N1—Ni1	116.62 (11)	N2—C15—C16	125.85 (16)
C15—N2—C14	118.61 (15)	N2—C15—H15A	117.1
C15—N2—Ni1	124.48 (12)	C16—C15—H15A	117.1
C14—N2—Ni1	116.51 (11)	C25—C16—C15	118.56 (15)
O1—C1—C10	124.15 (17)	C25—C16—C17	120.04 (16)
O1—C1—C2	116.95 (17)	C15—C16—C17	120.90 (16)
C10—C1—C2	118.90 (17)	C18—C17—C22	117.34 (16)
C3—C2—C1	120.88 (19)	C18—C17—C16	123.42 (16)
C3—C2—H2A	119.6	C22—C17—C16	119.22 (16)
C1—C2—H2A	119.6	C19—C18—C17	121.63 (19)
C2—C3—C4	122.0 (2)	C19—C18—H18A	119.2
C2—C3—H3A	119.0	C17—C18—H18A	119.2
C4—C3—H3A	119.0	C18—C19—C20	120.7 (2)
C9—C4—C5	119.5 (2)	C18—C19—H19A	119.6
C9—C4—C3	118.73 (17)	C20—C19—H19A	119.6
C5—C4—C3	121.8 (2)	C21—C20—C19	119.53 (19)
C6—C5—C4	120.9 (2)	C21—C20—H20A	120.2
C6—C5—H5A	119.6	C19—C20—H20A	120.2
C4—C5—H5A	119.6	C20—C21—C22	121.19 (19)
C5—C6—C7	119.9 (2)	C20—C21—H21A	119.4
C5—C6—H6A	120.0	C22—C21—H21A	119.4
C7—C6—H6A	120.0	C21—C22—C17	119.57 (18)
C8—C7—C6	120.6 (2)	C21—C22—C23	121.76 (18)
C8—C7—H7A	119.7	C17—C22—C23	118.68 (16)
C6—C7—H7A	119.7	C24—C23—C22	121.89 (17)
C7—C8—C9	121.3 (2)	C24—C23—H23A	119.1
C7—C8—H8A	119.3	C22—C23—H23A	119.1
C9—C8—H8A	119.3	C23—C24—C25	121.08 (18)
C8—C9—C4	117.77 (18)	C23—C24—H24A	119.5
C8—C9—C10	122.98 (19)	C25—C24—H24A	119.5
C4—C9—C10	119.22 (18)	O2—C25—C16	124.25 (16)
C1—C10—C11	118.64 (16)	O2—C25—C24	116.85 (16)
C1—C10—C9	120.05 (18)	C16—C25—C24	118.88 (16)
C11—C10—C9	120.84 (17)	C13—C26—H26A	109.5
N1—C11—C10	125.94 (17)	C13—C26—H26B	109.5
N1—C11—H11A	117.0	H26A—C26—H26B	109.5
C10—C11—H11A	117.0	C13—C26—H26C	109.5
N1—C12—C13	113.23 (14)	H26A—C26—H26C	109.5
N1—C12—H12A	108.9	H26B—C26—H26C	109.5
C13—C12—H12A	108.9	C13—C27—H27A	109.5
N1—C12—H12B	108.9	C13—C27—H27B	109.5
C13—C12—H12B	108.9	H27A—C27—H27B	109.5
H12A—C12—H12B	107.7	C13—C27—H27C	109.5
C27—C13—C14	110.24 (16)	H27A—C27—H27C	109.5
C27—C13—C12	107.57 (17)	H27B—C27—H27C	109.5
			
O2—Ni1—O1—C1	151.46 (15)	C9—C10—C11—N1	168.37 (17)
N1—Ni1—O1—C1	−30.88 (15)	C11—N1—C12—C13	114.41 (18)
O1—Ni1—O2—C25	151.58 (14)	Ni1—N1—C12—C13	−71.51 (17)
N2—Ni1—O2—C25	−30.68 (14)	N1—C12—C13—C27	155.32 (16)
O1—Ni1—N1—C11	24.19 (15)	N1—C12—C13—C14	35.0 (2)
N2—Ni1—N1—C11	−153.69 (15)	N1—C12—C13—C26	−83.63 (19)
O1—Ni1—N1—C12	−149.53 (12)	C15—N2—C14—C13	115.85 (17)
N2—Ni1—N1—C12	32.58 (12)	Ni1—N2—C14—C13	−71.04 (18)
O2—Ni1—N2—C15	23.74 (14)	C27—C13—C14—N2	−85.4 (2)
N1—Ni1—N2—C15	−154.06 (14)	C12—C13—C14—N2	33.4 (2)
O2—Ni1—N2—C14	−148.93 (12)	C26—C13—C14—N2	153.68 (16)
N1—Ni1—N2—C14	33.28 (12)	C14—N2—C15—C16	168.55 (16)
Ni1—O1—C1—C10	17.2 (2)	Ni1—N2—C15—C16	−4.0 (2)
Ni1—O1—C1—C2	−163.52 (13)	N2—C15—C16—C25	−18.9 (3)
O1—C1—C2—C3	176.67 (17)	N2—C15—C16—C17	169.17 (16)
C10—C1—C2—C3	−4.0 (3)	C25—C16—C17—C18	174.81 (17)
C1—C2—C3—C4	0.3 (3)	C15—C16—C17—C18	−13.4 (3)
C2—C3—C4—C9	2.0 (3)	C25—C16—C17—C22	−3.5 (2)
C2—C3—C4—C5	−178.28 (19)	C15—C16—C17—C22	168.25 (16)
C9—C4—C5—C6	−0.5 (3)	C22—C17—C18—C19	−1.5 (3)
C3—C4—C5—C6	179.7 (2)	C16—C17—C18—C19	−179.85 (18)
C4—C5—C6—C7	−0.8 (3)	C17—C18—C19—C20	0.4 (3)
C5—C6—C7—C8	0.7 (3)	C18—C19—C20—C21	0.3 (3)
C6—C7—C8—C9	0.8 (3)	C19—C20—C21—C22	0.0 (3)
C7—C8—C9—C4	−2.0 (3)	C20—C21—C22—C17	−1.1 (3)
C7—C8—C9—C10	−179.83 (18)	C20—C21—C22—C23	179.44 (19)
C5—C4—C9—C8	1.9 (3)	C18—C17—C22—C21	1.8 (2)
C3—C4—C9—C8	−178.36 (18)	C16—C17—C22—C21	−179.80 (16)
C5—C4—C9—C10	179.77 (17)	C18—C17—C22—C23	−178.73 (17)
C3—C4—C9—C10	−0.5 (3)	C16—C17—C22—C23	−0.3 (2)
O1—C1—C10—C11	12.5 (3)	C21—C22—C23—C24	−178.18 (18)
C2—C1—C10—C11	−166.77 (16)	C17—C22—C23—C24	2.3 (3)
O1—C1—C10—C9	−175.30 (16)	C22—C23—C24—C25	−0.5 (3)
C2—C1—C10—C9	5.5 (2)	Ni1—O2—C25—C16	17.6 (2)
C8—C9—C10—C1	174.51 (17)	Ni1—O2—C25—C24	−163.95 (12)
C4—C9—C10—C1	−3.3 (2)	C15—C16—C25—O2	11.7 (3)
C8—C9—C10—C11	−13.4 (3)	C17—C16—C25—O2	−176.29 (15)
C4—C9—C10—C11	168.79 (16)	C15—C16—C25—C24	−166.67 (16)
C12—N1—C11—C10	169.46 (16)	C17—C16—C25—C24	5.3 (2)
Ni1—N1—C11—C10	−4.1 (2)	C23—C24—C25—O2	178.13 (17)
C1—C10—C11—N1	−19.5 (3)	C23—C24—C25—C16	−3.3 (3)

### Hydrogen-bond geometry (Å, °)

**Table:**

*D*—H···*A*	*D*—H	H···*A*	*D*···*A*	*D*—H···*A*
C12—H12B···O1^i^	0.97	2.46	3.422 (2)	171
C14—H14B···O2^ii^	0.97	2.54	3.502 (2)	170

Symmetry codes: (i) −*x*+1, −*y*+2, −*z*; (ii) −*x*, −*y*+2, −*z*.
